# Protective properties of heme oxygenase-1 expressed in umbilical cord mesenchymal stem cells help restore the ovarian function of premature ovarian failure mice through activating the JNK/Bcl-2 signal pathway-regulated autophagy and upregulating the circulating of CD8^+^CD28^−^ T cells

**DOI:** 10.1186/s13287-019-1537-x

**Published:** 2020-02-04

**Authors:** Na Yin, Chenting Wu, Jianping Qiu, Yueming Zhang, Le Bo, Ying Xu, Mengdie Shi, Songyue Zhu, Guangzhao Yang, Caiping Mao

**Affiliations:** 1 Reproductive Medicine Center, The First Affiliated Hospital of Soochow University, 188 Shizi Rd, Suzhou, Jiangsu China; 2 Department of Gynaecology, The Affiliated Suzhou Municipal Hospital of Nanjing Medical University, Suzhou, Jiangsu China; 3 Department of Gynaecology and Obstetrics, The First Affiliated Hospital of Soochow University, Suzhou, Jiangsu China

**Keywords:** Heme oxygenase-1, Umbilical cord-derived mesenchymal stem cells, Premature ovarian failure, JNK/Bcl-2 signal pathway, Autophagy, CD8^+^CD28^−^ T cells

## Abstract

**Background:**

Umbilical cord-derived mesenchymal stem cell (UCMSCs) transplantation has been widely studied in premature ovarian failure (POF). However, the underlying mechanism remains elusive. This study aims to investigate the protective properties and mechanisms of heme oxygenase-1 (HO-1) expressed in UCMSCs in restoring the ovarian function of POF mice.

**Methods:**

In in vitro and in vivo experiments, mice were treated with the presence or absence of the HO-1/shHO-1-transfected UCMSCs, and the administration of SP600125 or anisomycin, the inhibitor or activator of JNK. The viability and apoptosis of granulosa cells (GCs) at different time points of co-cultivation were assessed in vitro. In in vivo experiments, mouse ovarian function was assessed by detecting the serum levels of hormone and observing the ovarian morphological changes. Multiple molecular indices of JNK/Bcl-2 signal pathway were performed. And the autophagy changes in GCs were assessed by detecting the associated cytokines and observing the intracellular autophagosome accumulation. Additionally, the spleen levels of CD8^+^CD28^−^ T cells and serum levels of interleukin 10 (IL-10) were tested to evaluate the immune mechanisms involved.

**Results:**

UCMSCs transfected with shHO-1 or treated with SP600125 inhibited GCs’ viability and promoted its apoptosis in a time-dependent manner in vitro. In in vivo experiments, mice in both groups showed little therapeutic efficiency which presented as the increased extent of ovarian fibrosis with decreased number of functional follicles, and disordered hormone production. Additionally, the JNK/Bcl-2-associated cytokines were obviously declined. The inhibited autophagy-related cytokines, the chromatin condensation and abound vacuolar autophagosome in GCs, and weakened fluorescence intensity by MDC were observed. The downregulated levels of CD8^+^CD28^−^ T cells and serum levels of IL-10 were also detected. The damages above can be alleviated with HO-1-MSCs treatment or anisomycin administration.

**Conclusions:**

HO-1 expressed in UCMSCs is critical in restoring the ovarian function in POF mice with UCMSC transplantation, which is mediated by the activation of JNK/Bcl-2 signal pathway-regulated autophagy and upregulating the circulating of CD8^+^CD28^−^ T cells.

## Background

As a heterogeneous disorder, premature ovarian failure (POF) is prevalent in 1–3% of women under 40 years old, and its features include the disordered ovarian function, the elevated gonadotropin hormone, and descended estrogen levels [1]. However, the more efficient therapeutic methods remain to be explored. With the ability of directionally differentiating into specific cells under specific microenvironments, secreting paracrine cytokines, regulating inflammation, and enhancing endogenous tissue repair [2], increasing evidences have proposed the transplantation of mesenchymal stem cells (MSCs) to be a potential therapy of treating POF disorder [3, 4]. Additionally, with the characteristics of easy extraction and low immunogenicity, human umbilical cord-derived mesenchymal stem cells (UCMSCs) have been recognized as the preferred MSCs for transplantation [5].

Heme oxygenase-1 (HO-1) expressed in most cells, which has potent anti-inflammatory, antioxidant, and immunoregulatory properties [6]. Researches have illustrated that MSCs can upregulate the expression of HO-1, which plays a critical therapeutic role [7]. It also participates in the physiology of the ovary [8] and the secretion of gonadotropins from the pituitary gland [9]. It has been proved that in various disease conditions, HO-1 induction is an adaptive defense mechanism to maintain the function of cells and tissues [10, 11]. However, much less is comprehended of the role and mechanism of HO-1 in recovering the ovarian function of POF mice with UCMSC transplantation. Attentions have been drawn to the HO-1-mediated autophagy pathway [12, 13]. In physical conditions, autophagy maintains cellular homeostasis [14–16], while under short-term stress, it promotes survival and viability [17]. In the ovary, autophagy in murine newborns participates in preserving the primordial oocyte pool [18] and is relevant to oocyte elimination during follicular atresia [19]. Researches showed that autophagy can be activated and weakened the restrain of Bcl-2 on Beclin-1 through activating the JNK/Bcl-2 signal pathway [20]. Moreover, autophagy is recognized to have participated into both innate and adaptive immunity [21].

CD8^+^ regulatory T (Treg) cells are known essential in various inflammatory disorders and autoimmune diseases [22]. As an important subset of CD8^+^Tregs, CD8^+^CD28^−^ T cells display typical immunosuppressive function [23]. Furthermore, evidences suggest that CD8^+^CD28^−^ T cells can induce immunological tolerance in transplantation, demonstrating immunologic suppression in organ transplants [24]. The expanding of the cells may be the mechanism of primary tolerance in bone morrow transplantation, improving the acceptability of graft and stabilizing its function, and its enhanced expansion is the reason of reduced immune rejection [25, 26]. To date, the altered expression of CD8^+^CD28^−^ T cells have been observed in many disease, including autoimmunity and chronic inflammatory disease [25, 27]. However, whether and how the UCMSC transplantation effect the expression of the CD8^+^CD28^−^ T cells should still be explored.

In this report, the HO-1/shHO-1 gene was incorporated into UCMSCs through plasmid transduction, aiming to explore the role and mechanism of HO-1 gene in recovering ovarian function of POF mice with UCMSC transplantation. And the research will supply helpful information in treating POF patients with MSCs transplantation in the clinic.

## Materials and methods

### Animals

Six-week-old female mice (for researches in vivo) and 3-week-old female mice (for researches in vitro) (C57BL/6) were purchased from Suzhou Zhaoyan Biotechnology Co. (Jiangsu, China). All the animals were housed and fed with proper diet.

### Isolation, culture, and identification of human UCMSCs and mouse ovarian granular cells (GCs)

Human umbilical cords were acquired from parturient woman with negative infectious diseases, and the written and informed consent was obtained for the umbilical cord sample will be used for research purpose. The umbilical cords were washed and cut into small pieces (1 mm^3^) without the blood vessels and placed onto plates with culture medium and maintained at 37 °C. The media were renewed at intervals of 2–3 days. To identify the UCMSCs, cell morphology was observed under inverted fluorescence microscope (Nikon, Japan). For osteogenic and adipogenic differentiation, alizarin red staining and Oil Red O staining (Cyagen, USA) were used separately to identify osteoblast-like cells and adipose cells. Additionally, the molecular markers of UCMSCs such as CD14, CD29, CD34, CD90, CD31, and HLA-DR were examined using FCM [28]. Cells used in the experiments were after three passages.

The pregnant mare serum gonadotropin (Solarbio, CN) were injected into 3-week-old female mice (i.p.), and mouse ovaries were extracted with peripheral adhesion tissues removed under sterile conditions 48 h later. To obtain single-cell suspension, the ovaries were washed and the GCs were released from follicles under an anatomical microscope. After washing with PBS, the cells were cultured at 37 °C, 5% CO_2_. First passage of GCs was used in the experiments. To identify the GCs, the expression of follicle-stimulating hormone receptor (FSHR) was detected by immunohistochemistry. After fixation and penetration, GCs were incubated overnight at 4 °C with rabbit anti-mouse FSHR antibody (1:150, proteintech, CN). And the secondary biotinylated goat anti-rabbit lgG antibody (1:300, Beyotime, CN) were used, following with DAB dye liquor. The dyeing conditions were recorded with inverted fluorescence microscope [4].

### Modification of HO-1 gene in UCMSCs via plasmid transfection

Before the transfection of HO-1/shHO-1(used to knock down HO-1 levels)/NC (empty vector) gene described by the manufacturer (Ribobio, CN), 2 × 10^5^ cells/well of UCMSCs were added into the 24-well transwell plates for 24 h. A non-targeting RNA was used as control, and two targeting RNAs were used in the transfection. Briefly, UCMSCs were incubated with plasmid for 24 h at 37 °C. And the culture medium was changed to incubate another 2 days. After RNAs and of Lipofectamine 3000 (Invitrogen, USA) were separately mixed with serum-free DMEM, the diluents were mixed and incubated at room temperature (RT) for 20 min, then were added into the cells and cultured for 72 h at 37 °C, 5% CO_2_. The transfection efficiency of HO-1 was investigated by the quantitative reverse-transcription polymerase chain reaction (qRT-PCR).

### UCMSC and GC transwell co-culture

The co-culture of UCMSCs and GCs was conducted based on the methods reported [29]. Briefly, 1 × 10^5^ cells/well of UCMSCs were seeded onto 24-well transwell permeable support (pore size, 0.4 μm, Corning, NY, USA), and cultured overnight at 37 °C, 5% CO_2_. Then, 5 × 10^4^ cells/well of GCs were seeded into the bottom of 24-well plates. After co-cultivation for 24 h, the following experiments were operated.

### Measurement of GCs’ viability by Cell Counting Kit-8 (CCK-8) analysis

In in vitro experiments, 5 mg/L chlormethine hydrochloride (HN2) were used to simulate POF model. The GCs’ viability was assessed according to the manufacturer’s instructions (MCE, USA). Briefly, 5 × 10^3^ cells/well of GCs were seeded in triplicate in 96-well plates and were co-cultured with the supernatant of UCMSCs transfected with the plasmids of HO-1/shHO-1/NC for 24 h prior to exposure to SP600125 (0–20 μM) or anisomycin (0–20 μM) for 12 h, 24 h, 48 h, and 72 h, respectively. Then, the medium was removed with fresh medium containing 10 μl of CCK-8 reagent added and incubated at 37 °C for 2 h. Absorbance (λ/nm = 450) was measured using a SpectraMax M2e microplate reader (Molecular Devices, USA). The GCs’ viability shows as the ratios of the OD values in the treated groups to the control group.

### Apoptosis assay by FCM

The FITC Annexin V apoptosis detection kit (Beyotime, CN) was used to assess the GC apoptosis. After collecting, washing the GCs, 500 μl 1× binding buffer mixed with 5 μl of Annexin-V-fluorescein isothiocyanate (FITC) and 10 μl of propidium iodide (PI) were added and detected using FCM (BD, USA).

### MDC staining

According to manufacturer’s protocols, 5 × 10^3^ cell/well of GCs were incubated in 96-well plates and treated with HN2 for 12 h and then were co-cultured with the supernatant of UCMSCs transfected with the plasmids of HO-1/shHO-1/NC for 24 h prior to exposure to SP600125 or anisomycin, respectively. After 48-h treatment, 100 μM of MDC was added for 15–60 min. After washing with 100 μl wash buffer, optical density was calculated by the inverted fluorescence microscope and analyzed by Image J software.

### Animal model establishment

Six-week-old mice (*n* = 81) were randomly divided into nine groups (*n* = 9): control group (A), POF group (B), POF+ UCMSCs group (C), POF+ NC-UCMSCs group (D), POF+ HO-1-UCMSCs group (E), POF+ shHO-1-UCMSCs group (F), POF+ UCMSCs+ DMSO group (G), POF+ UCMSCs+ SP600125 group (H), and POF+ UCMSCs+ Anisomycin group (I). Mice in group A received no treatments. Mice in groups B–I were injected i.p. with cyclophosphamide (120 mg/kg) and busulfan (30 mg/kg) [30]. 1 × 10^6^ MSCs of 6th passages were suspended with PBS and injected into mice in groups C, G, H, and I, and MSCs transfected with NC, HO-1, or shHO-1 gene were separately injected into mice in groups D, E, and F 2 weeks later, according to the previous studies [31–33]. Another 5 days later, mice in groups H and I were treated with SP600125 (MCE, USA, 15 mg/kg, i.p.) or anisomycin (Selleck, USA, 5 mg/kg, i.p.), respectively. In mice of group G, 0.01% DMSO were treated (i.p.) as a vehicle control of groups H and I. Then 48 h later, all mice were sacrificed to do the following experiments.

### Serum levels of hormone and interleukin (IL-10) measurement

Blood samples of mice were taken from postcava and centrifuged to get the serum. The ELISA kits (Greenleaf, CN) were used to survey the levels of estradiol (E_2_), follicle stimulation hormone (FSH), luteinizing hormone (LH), anti-Müllerian hormone (AMH), and IL-10 according to the instructions of manufacturers.

### Follicle counting and ovarian morphological analysis

Before staining with HE for histopathology, mouse ovaries were kept and fixed. The ovarian histological changes were observed by light microscopy (Olympus). We counted the follicles containing obvious nucleus in the experiment, which were divided into primordial, primary, secondary, antral, and atretic follicles, which were described before [3, 34].

### QRT-PCR

The RNeasy Mini Kits (Qiagen, Germany) were used to extract RNA from mouse ovaries based on the manufacturer’s protocols. To generate cDNA, 1 μg of RNA with oligo dT and a reverse transcription kit (Transgen Biotech, CN) was used. The primers are presented in Table 1, and FastStart Universal SYBR Green Master (Thermo Fisher Scientific, USA) were used to carry quantitative PCR with the StepOnePlus™ Real-Time PCR System (Thermo Fisher Scientific, USA). GAPDH was used for control. Each sample was tested three times [3].

**Table 1:** Sequences of the primers used in QRT-PCR

Target gene	Primer	Nucleotide sequence
*h-GAPDH*	F	5′-GGAGCGAGATCCCTCCAAAAT-3′
	R	5′-GGCTGTTGTCATACTTCTCATGG-3′
*h-HO-1*	F	5′-AAGACTGCGTTCCTGCTCAAC-3′
	R	5′-AAAGCCCTACAGCAACTGTCG-3′
*m-GAPDH*	F	5′-AGGTCGGTGAACGGATTTG-3′
	R	5′-TGTAGACCATGTAGTTGAGGTCA-3′
*m-BCL-2*	F	5′-GCTACCGTCGTGACTTCGC-3′
	R	5′-CCCCACCGAACTCAAAGAAGG-3′
*m-Beclin1*	F	5′-ATGGAGGGGTCTAAGGCGTC-3′
	R	5′-TCCTCTCCTGAGTTAGCCTCT-3′
*m-p62*	F	5′-AGGATGGGGACTTGGTTGC-3′
	R	5′-TCACAGATCACATTGGGGTGC-3′
*m-LC3I/II*	F	5′-GACCGCTGTAAGGAGGTGC-3′
	R	5′-CTTGACCAACTCGCTCATGTTA-3′
*m-Atg5*	F	5′-TGTGCTTCGAGATGTGTGGTT-3′
	R	5′-GTCAAATAGCTGACTCTTGGCAA-3′
*m-HO-1*	*F*	*5′-AAGCCGAGAATGCTGAGTTCA-3*′
	*R*	*5′-GCCGTGTAGATATGGTACAAGGA-3*′

### Western blotting

Ovaries were lysed by radioimmunoprecipitation assay (RIPA) buffer. After measuring the concentration of the protein, 10% sodium dodecyl sulfatepolyacrylamide gel electrophoresis (SDS-PAGE) gel electrophoresis was used to separate and transferred to the membrane, which was blocked with serum blocking solution, and then incubated overnight at 4 °C with primary antibodies against JNK, p-JNK, and HO-1 (1:1000, Proteintech, USA); Bcl-2, Beclin-1, Atg5, p62, and LC3I/II (1:1000, CST, USA); and GAPDH (1:50000, Proteintech). After three times of washing, the secondary antibodies were added to the membrane and incubated for 1 h at RT, then detected using the Super Enhancer chemiluminescence (ECL) Kit (Absin, CN). Results were analyzed by Image J software.

### Transmission electron microscopy (TEM)

The ovaries were rinsed twice with PBS and post-fixed in 1% osmium tetroxide (OsO 4) at 4 °C overnight; cells were dehydrated in graded alcohols, embedded in epoxy resins, sectioned and stained with uranyl acetate/lead citrate, and observed with TEM (FEI Tecnai Spirit, USA). Each group was prepared in three sections, and 200 cells were used to observe the autophagic structures.

### Differentiation of CD8^+^CD28^−^ T lymphocytes by FCM

Mice spleens were mechanically minced. After lysing with lymphocyte separation medium, cells were resuspended in PBS. With 10-min incubation of anti-mouse CD3 APC, anti-mouse CD8 FITC, and anti-mouse CD28 PE (eBioscience, San Diego, USA) at 4 °C in the dark, the cell suspension was analyzed by FCM.

### Data analysis

SPSS 16.0 software was applied to analyze the data for thrice in each experiment, which are then shown as the mean ± standard deviation. The Student *t* test was analyzed to compare each of the two groups. The distribution of data was analyzed by a one-way analysis of variance (ANOVA). *P* value of < 0.05 refers statistically significant.

## Results

### The primary culture of UCMSCs and GCs, and the transduction efficiency of UCMSCs with HO-1/shHO-1/NC plasmids

**Fig. 1:**
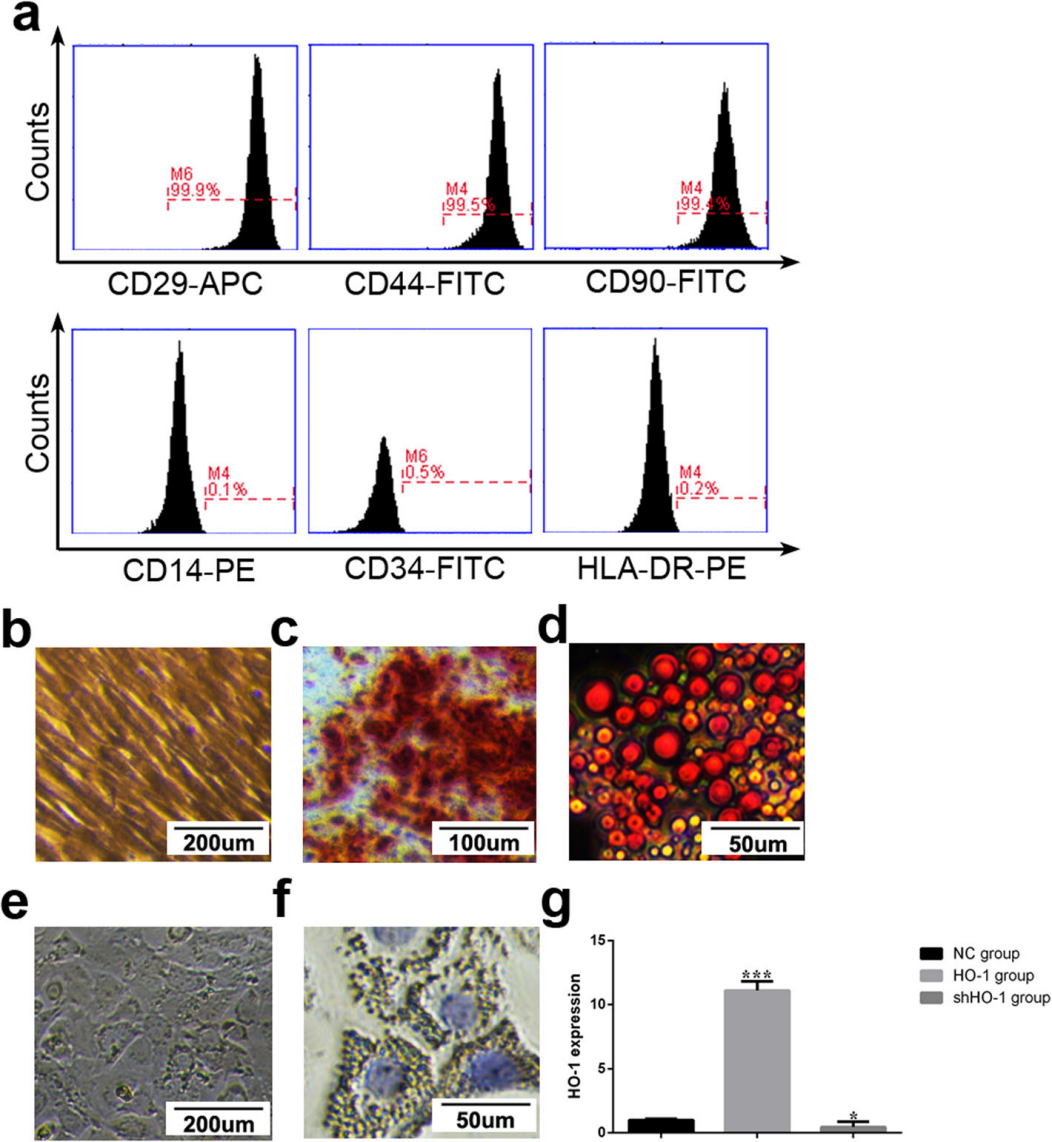
Identification of UCMSCs and GCs, and the transduction efficiency of the HO-1/shHO-1 plasmids into UCMSCs. **a** Black histograms represent expression of indicated cell surface marker. **b** Cultured UCMSCs show fibroblast-like morphology (× 100). **c**, **d** UCMSCs cultured under conditions for differentiation into osteoblasts or lipoblasts. Osteoblasts are displayed by Alizarin Red staining and darker red staining indicates calcium deposition (× 200, **c**). Lipoblasts displayed by accumulation of neutral lipid vacuoles stained with Oil Red O (× 400, **d**). **e**, **f** Morphology and phenotypes of GCs. Cultured GCs show spindle-shaped morphology (× 100, **e**). Blue staining indicates the GCs’ nucleus; brown staining indicates FSHR-positive expression in cytoplasm (× 400, **f**). **g** The transfection efficiency of the HO-1/shHO-1 plasmids into UCMSCs. *P < 0.05, ***P < 0.001 vs NC group. GCs, granulosa cells; HO-1, heme oxygenase-1; UCMSCs, umbilical cord mesenchymal stem cells

Individual clone spheres were formed until 7–10 days after inoculation and displayed fibroblast-like morphology (Fig. 1b). Stable cell population can be observed three passages later, and no visible morphologic alteration was observed even following 10 passages. Positive expression of CD29, CD44, and CD90 were detected with the immunophenotyping analysis. And the negative expression of CD34, CD14, and HLA-DR were detected (Fig. 1a). In osteoblastic induction medium, von Kossa staining showed calcium deposition (Fig. 1c). In adipogenic induction medium, and Oil Red O staining to observe the lipid droplets in the cytoplasm was positive (Fig. 1d), which were consistent with the researches published [35]. A significantly higher expression levels of HO-1mRNA were observed in the HO-1 plasmid transduction group (*P* < 0.001), and lower expression was in the shHO-1 plasmid transduction group comparing with the NC transduction group (*P* < 0.05) (Fig. 1g), which represents that the HO-1/shHO-1 plasmids have been effectively transfected into UCMSCs.

Cells isolated from mouse ovarian follicles were observed as adherent growth after 24 h of inoculation and displayed polygon-like morphology (Fig. 1e). FSHR, which can serve as a marker of GCs, is positive in almost all of the cells’ cytoplasm (Fig. 1f), in accordance with our previous reports [3].

### HO-1 expressed in UCMSCs increased GCs’ viability and decreased their apoptosis overtime

**Fig. 2:**
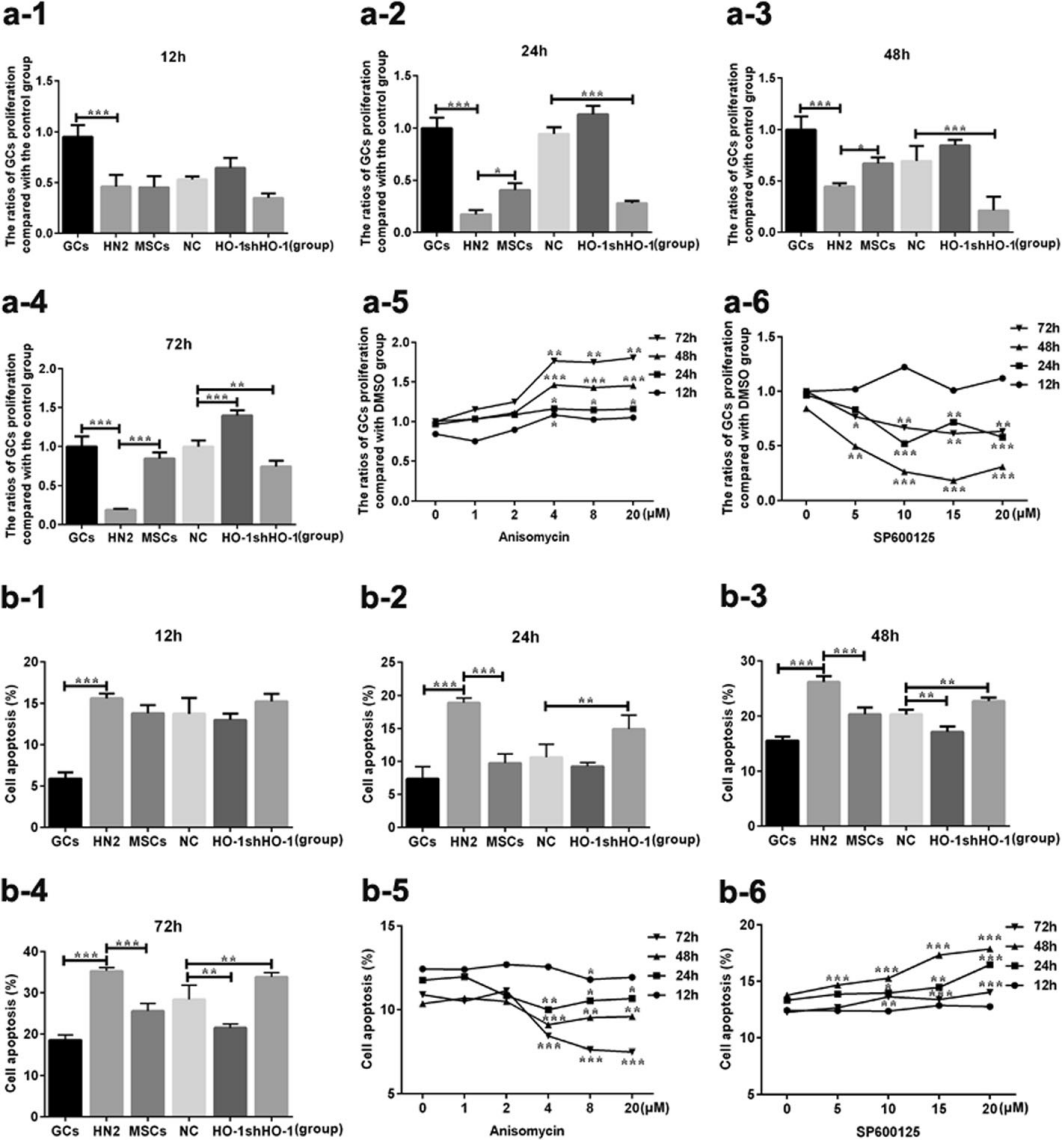
The proliferation and apoptosis of GCs in different condition. The statistical charts of GCs viability co-cultured with UCMSCs transfected with the plasmids of HO-1/shHO-1 for 12 h (**a-1**), 24 h (**a-2**), 48 h (**a-3**), or 72 h (**a-4**). The tendency charts of GC proliferation administrated with anisomycin (0–20 μM) (**a-5**) or SP600125 (0–20 μM) for 12–72 h (**a-6**). The statistical charts of GCs’ apoptosis co-cultured with UCMSCs transfected with the plasmids of HO-1/shHO-1 for 12 h (**b-1**), 24 h (**b-2**), 48 h (**b-3**), or 72 h (**b-4**). The tendency charts of GC apoptosis administrated with anisomycin (0–20 μM) (**b-5**), or treated with SP600125 (0–20 μM) for 12–72 h (**b-6**). Data presented as mean ± SD. *P < 0.05, **P < 0.01, ***P < 0.001 vs DMSO group, GC group, or NC group, separately. DMSO, dimethylsulfoxide; GCs, granulosa cells; HO-1, heme oxygenase-1; NC, empty vector; UCMSCs, umbilical cord mesenchymal stem cells

**Fig. 3:**
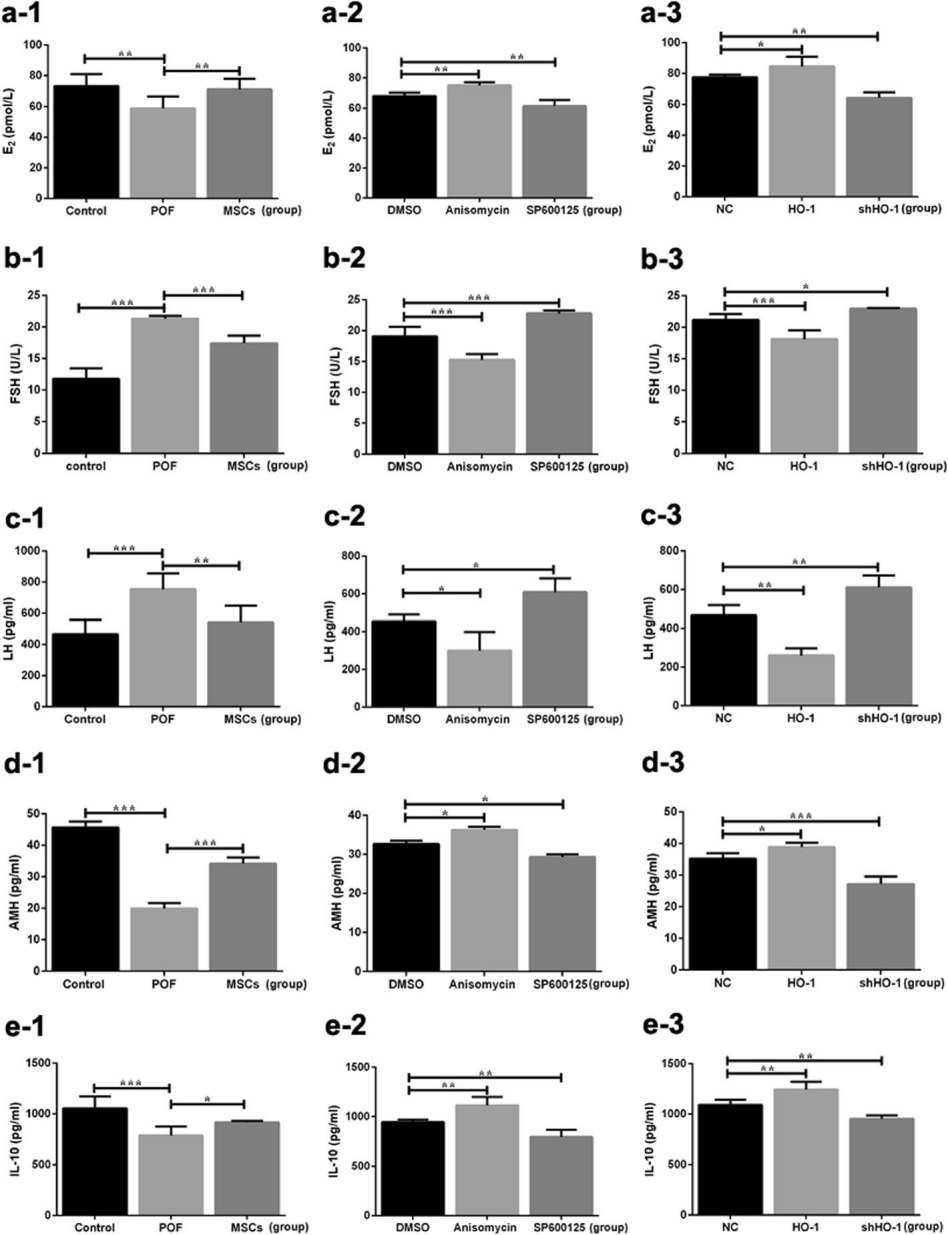
Serum levels of E_2_, FSH, LH, AMH, and IL-10 in mice. **a-1**–**a-3** E_2_ release. **b-1**–**b-3** FSH release. **c-1**–**c-3** LH release. **d-1**–**d-3** AMH release. **e-1**–**e-3** IL-10 release. Data presented as mean ± SD. *P < 0.05, **P < 0.01, ***P < 0.001 vs DMSO, GC group, or NC group, separately. DMSO, dimethylsulfoxide; E_2_, estradiol; FSH, follicle stimulation hormone; HO-1, heme oxygenase-1; IL-10, interleukin-10; LH, luteinizing hormone; POF, premature ovarian failure; UCMSCs, umbilical cord mesenchymal stem cells

**Fig. 4:**
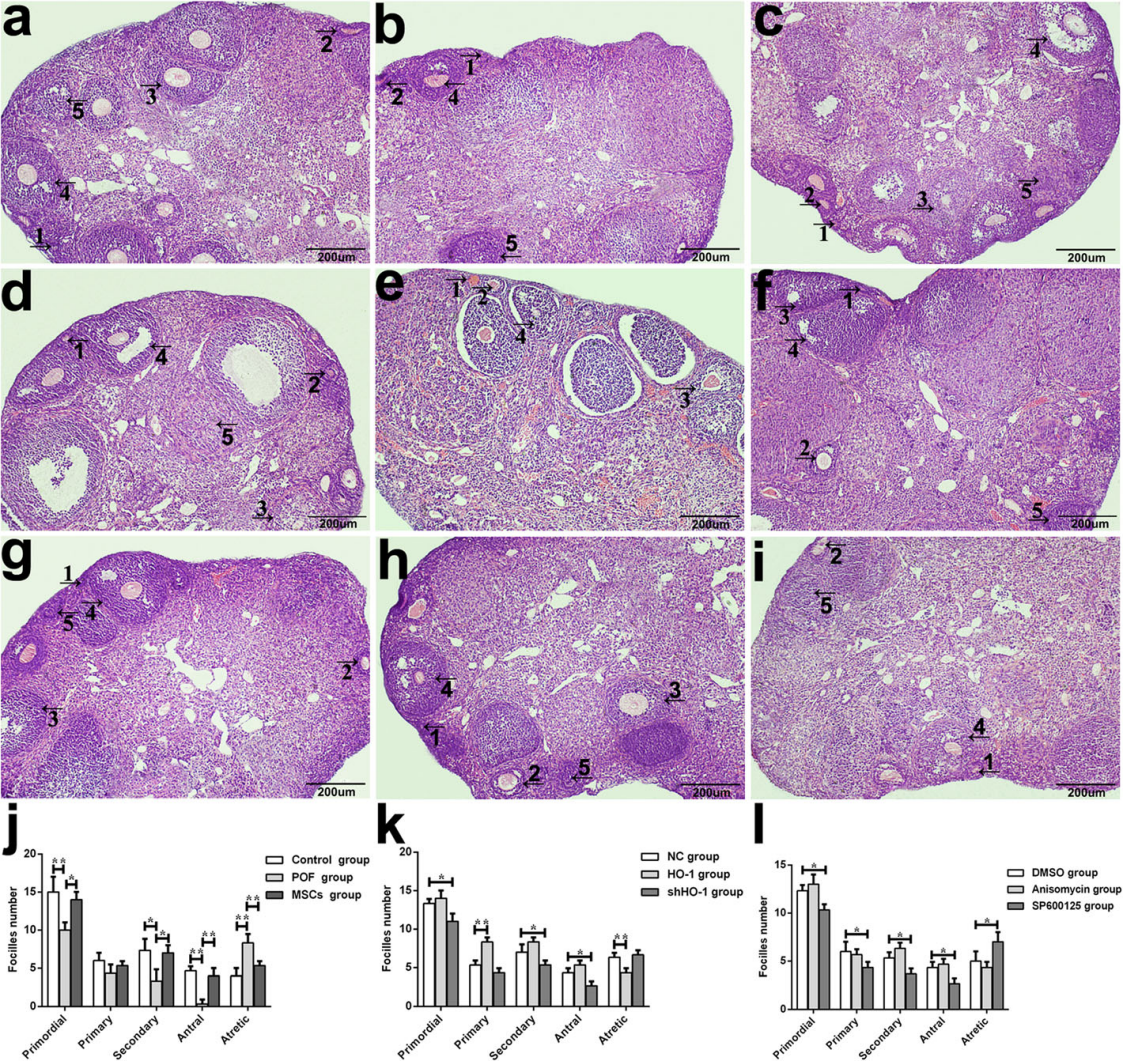
Histopathological examination of ovarian tissues. Photomicrographs (× 100) show HE stained ovaries. **a** Control group. **b** POF group. **c** MSC group. **d** NC group. **e** HO-1 group. **f** shHO-1 group. **g** DMSO group. **h** Anisomycin group. **i** SP600125 group. The five types of ovarian follicles were marked as (1) primordial follicles, (2) primarily follicles, (3) secondary follicles, (4) antral follicles, and (5) atresia follicles. **j**–**l** Quantitation on follicle count from ovaries in mice of the nine groups. Data presented as mean ± SD. *P < 0.05, **P < 0.01, ***P < 0.001 vs DMSO, GC group, or NC group, separately. Bar scale = 200 μm. DMSO, dimethylsulfoxide; HE, hematoxylin and eosin; HO-1, heme oxygenase-1; NC, empty vector; POF, premature ovarian failure; UCMSCs, umbilical cord mesenchymal stem cells

HO-1 expressed in UCMSCs induced GCs’ viability in a time-dependent manner (Fig. 2). All the viabilities in the treatment groups were significantly decreased compared with the GC group (*P* < 0.001) (Fig. 2a-1). To the time point of 24 h, the decreased viability in GCs treated with HN2 was significantly increased with the co-cultivation of MSCs or HO-1-MSCs, while with the co-cultivation of shHO-1-MSCs, the GCs’ viability showed lower than that in the NC group (*P* < 0.001) (Fig. 2a-2), which were similar to the tendency in 48 h (Fig. 2a-3). To the time point of 72 h, upregulated levels of the viability were detected in the HO-1 group than the NC group (*P* < 0.001), and the downregulated levels of viability was still presented in the shHO-1 group (*P* < 0.01) (Fig. 2a-4).

As expected, the GCs’ apoptosis was reduced over time with the co-cultivation of HO-1-UCMSCs (Fig. 2). Similar to the results presented in 12 h, no significant differences were detected among the treatment groups (*P* > 0.05), while it was obviously upregulated than that in 12 h (*P* < 0.001) (Fig. 2b-1). While to the time point of 24 h, the increased GCs’ apoptosis in the HN2 group can be reversed by treating with MSCs, and the apoptosis in the shHO-1 group was detected higher than the NC group (*P* < 0.01) (Fig. 2b-2). When it turns to 48 h, the apoptosis of GCs in the HO-1 group presented as significantly lower than that in the NC group (*P* < 0.01), while higher apoptosis still presented in the shHO-1 group (*P* < 0.01) (Fig. 2b-3), and similar tendencies can be observed when it turns to 72 h (Fig. 2b-4). From these tendencies, it can be preliminary indicated that HO-1 expressed in UCMSCs played an essential role in the therapeutic process of POF mice receiving UCMSC transplantation.

### HO-1 expressed in UCMSCs help recover the ovarian function in POF mice

To further confirm the critical role of HO-1 expressed in the UCMSCs during the therapeutic process, the HO-1/shHO-1-MSCs were transplanted into POF mice and the ovarian function were analyzed. Results recognized that comparing with the control group, decreased levels of E_2_ (*P* < 0.01) and AMH (*P* < 0.001) and increased levels of FSH (*P* < 0.001) and LH (*P* < 0.001) were detected in the POF group, which can be reversed by the administration of MSCs (Fig. 3a-1, b-1, c-1). Similarly, the uptrend levels of E_2_ (*P* < 0.05), AMH (*P* < 0.05) and downtrend levels of FSH (*P* < 0.001) and LH (*P* < 0.01) can be observed in the HO-1 group, while decreased levels of E_2_ (*P* < 0.01), AMH (*P* < 0.001) and increased levels of FSH (*P* < 0.05) and LH (*P* < 0.01) were detected in the shHO-1 group compared with those in the NC group, which represents the obviously weakened therapeutic efficiency (Fig. 3a-3, b-3, c-3).

**Fig. 5:**
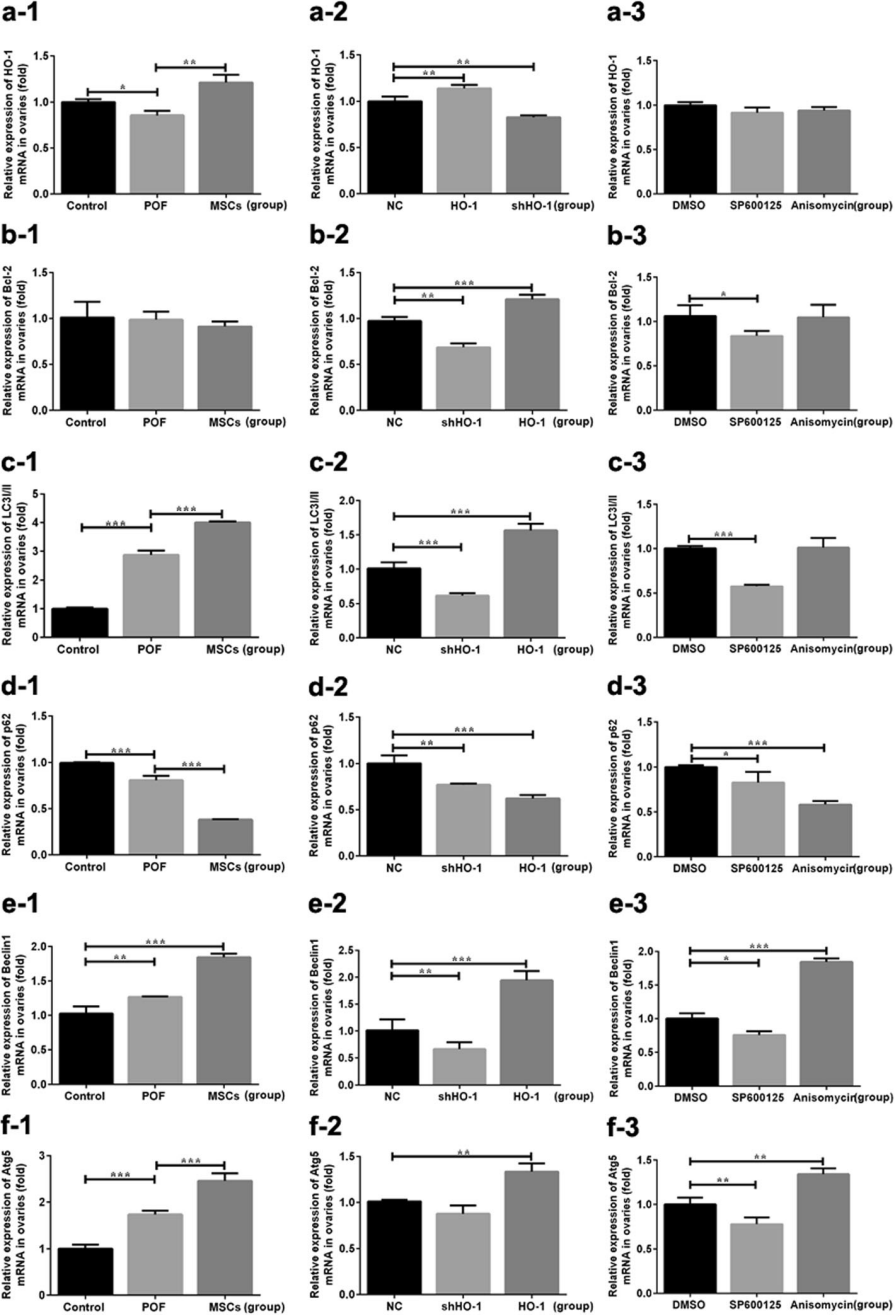
HO-1, Bcl-2, and autophagy-related mRNA expression in ovarian tissues by QRT-PCR. **a-1**–**a-3** HO-1 mRNA expression. **b-1**–**b-3** Bcl-2 mRNA expression. **c-1**–**c-3** LC3II/I mRNA expression. **d-1**–**d-3** p62 mRNA expression. **e-1**–**e-3** beclin1 mRNA expression. **f-1**–**f-3** Atg5 mRNA expression. *P < 0.05, **P < 0.01, ***P < 0.001 vs DMSO, GC group, or NC group, separately. DMSO, dimethylsulfoxide; HO-1, heme oxygenase-1; NC, empty vector; POF, premature ovarian failure; UCMSCs, umbilical cord mesenchymal stem cells

Large numbers of different stages of healthy follicles were contained in mice ovaries of the control group, including primordial follicles (Fig. 4-1), primary follicles (Fig. 4-2), secondary follicles (Fig. 4-3), antral follicles (Fig. 4-4), and atretic follicles (Fig. 4-5). However, it presented as the atrophied ovaries in the POF group which showed serious fibrosis with a reduced number of function follicles (Fig. 4b), which showed the similar morphology to the shHO-1 group (Fig. 4f). With the MSC or HO-1-MSC administration, the morphology of ovaries gradually return to normal (Fig. 4c, e). Additionally, similar to the declined functional follicles (*P* < 0.05) and inclined atretic follicles (*P* < 0.01) in the POF group compared with the control group, a declined trend of primordial follicles (*P* < 0.05), secondary follicles (*P* < 0.05), and antral follicles (*P* < 0.05) were showed in the shHO-1 group comparing with the NC group (Fig. 4j, k). With the transfection of HO-1, the primary follicles were obviously upregulated (*P* < 0.05), along with downregulated number of the atretic follicles (*P* < 0.01). Based upon the results above, we can further confirm that HO-1 gene played a critical role during the therapeutic process of POF mice receiving MSC transplantation.

### HO-1 expressed in UCMSCs help recover the ovarian function of POF mice through activating the JNK/Bcl-2 signal pathway

**Fig. 6:**
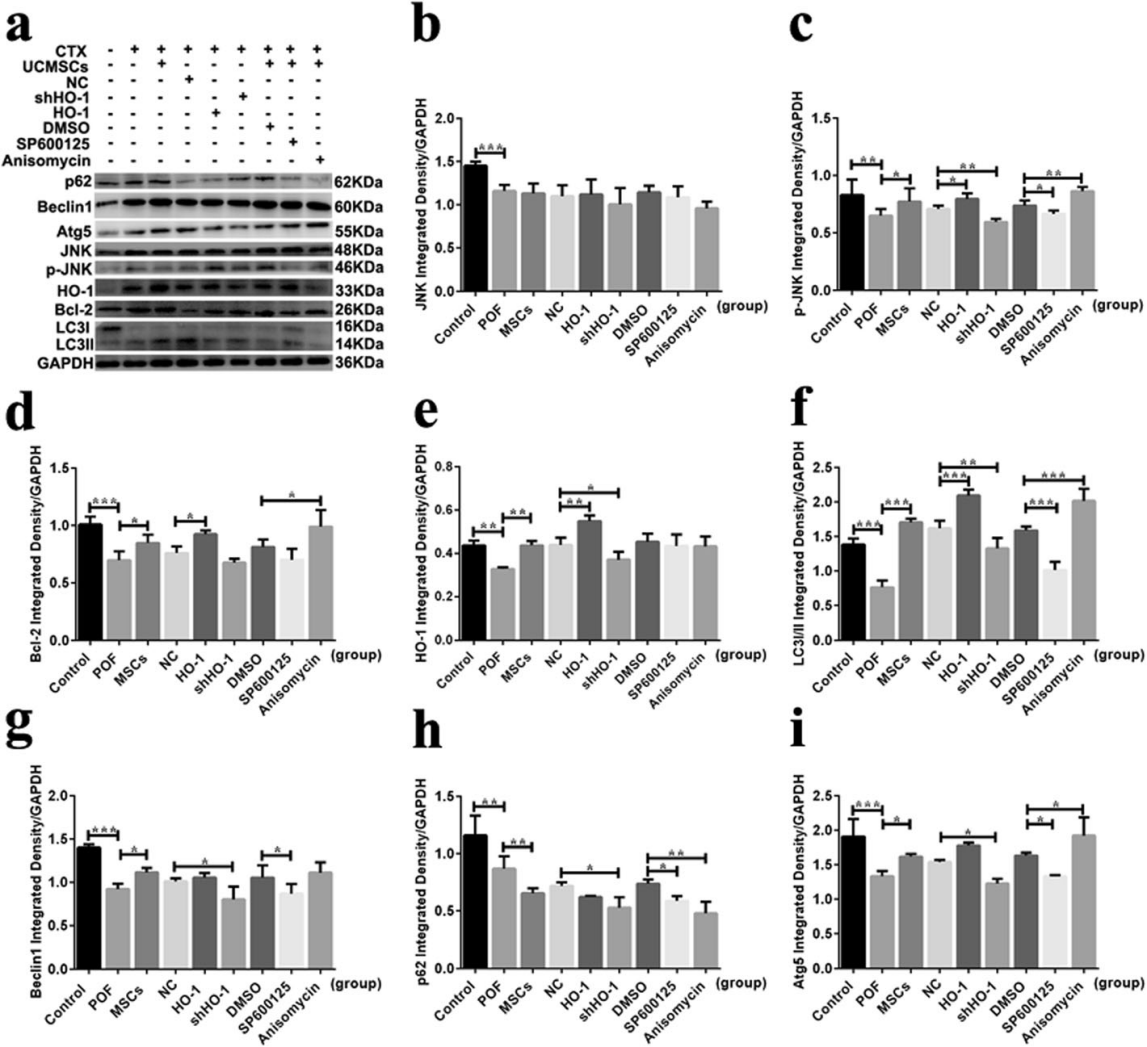
HO-1, JNK/Bcl-2 signal pathway- and autophagy-related protein expression in ovarian tissues by western blot analysis. **a** The HO-1, JNK/Bcl-2 signal pathway- and autophagy-related protein expression in PVDF membrane. Quantification of **b** JNK, **c** p-JNK, **d** Bcl-2, **e** HO-1, **f** LC3II/I, **g** Beclin1, **h** p62, and **i** Atg5 protein expression. *P < 0.05, **P < 0.01, ***P < 0.001 vs DMSO, GC group, or NC group, separately. DMSO, dimethylsulfoxide; HO-1, heme oxygenase-1; NC, empty vector; POF, premature ovarian failure; UCMSCs, umbilical cord mesenchymal stem cells

To figure out whether and how the JNK/Bcl-2 signal pathway involved in the therapeutic process, in vivo and in vitro experiments were operated with or without the inhibitor or activator of the JNK/Bcl-2 signal pathway. In in vitro experiments, the GCs’ viability significantly declined with the administration of SP600125 in a time- and dose-dependent manner, observed at the concentrations below 15 μM to the time points of 48 h (Fig. 2a-6). Similarly, the GCs’ viability was promoted by the treatment of anisomycin at the concentration below 4 μM to the time points of 48 h (Fig. 2a-5). To further confirm the changes above, the apoptosis of GCs was analyzed. Decreased tendency of GCs’ apoptosis was observed with the treatment of anisomycin (Fig. 2b-5), while the usage of SP600125 can obviously increase the apoptosis as the dose and time increased (Fig. 2b-6). In in vivo experiments, the serum levels of E_2_ and AMH were inclined (*P* < 0.01 and *P* < 0.05) with the administration of anisomycin, but the levels of FSH (*P* < 0.001) and LH (*P* < 0.01) were declined compared with the DMSO group (Fig. 3). With the treatment of SP600125, the serum levels of E_2_ were and AMH were downregulated (*P* < 0.01 and *P* < 0.05) but the levels of FSH (*P* < 0.001) and LH (*P* < 0.05) were upregulated. In the aspect of morphology, shown in Fig. 4, the number of follicles in each stage showed the similar tendency with that in the anisomycin group. Meanwhile in the SP600125 group, mouse ovaries displayed as reduced number of functional follicles (*P* < 0.05), along with increased number of atretic follicles (*P* < 0.05).

To elucidate whether HO-1-MSCs played its role through activating the JNK/Bcl-2 signal pathway during the therapeutic process, the expression levels of HO-1 and the JNK/Bcl-2 signal pathway-related proteins and mRNA were detected. The HO-1 mRNA expression levels in the HO-1 group showed significantly upregulated compared with that in the NC group (*P* < 0.01), while it presented as downregulated in the shHO-1 group (*P* < 0.01). The expression levels of Bcl-2 mRNA were declined in the shHO-1 group (*P* < 0.01) but inclined in the HO-1 group (*P* < 0.001) compared with the NC group (Fig. 5b-2), and with the administration of SP600125, it showed significantly decreased compared with the DMSO group (*P* < 0.05, Fig. 5b-3). Corresponding to the tendencies above, the HO-1 protein levels were significantly increased in the HO-1 group when compared with the NC group (*P* < 0.01), while the tendency showed decreased in the shHO-1 group (*P* < 0.05, Fig. 6e). No significant changes were observed among the groups except the control group in JNK protein expression (*P* > 0.05, Fig. 6b). However, the p-JNK protein expressed in the HO-1 group were significantly increased (*P* < 0.01) but decreased obviously in the shHO-1 group (*P* < 0.01) compared with the NC group. With the administration of anisomycin, the expression levels of p-JNK were increased (*P* < 0.01), while the treatment of SP600125 can decrease the levels (*P* < 0.05, Fig. 6c). Increased levels were detected in the HO-1 group (*P* < 0.05), but no significant changes were observed in the shHO-1 group (*P* > 0.05), and the inclined tendency was presented in the anisomycin group (*P* < 0.05, Fig. 6d). Based on the results above, we can suggest that the ovarian function of POF mice receiving MSC transplantation may be restored through activating the JNK/Bcl-2 signal pathway.

### HO-1 expressed in UCMSCs induce robust autophagic flux through activating the JNK/Bcl-2 signal pathway in the therapeutic process

To further confirm whether and how the activating of JNK/Bcl-2 signal pathway regulates autophagy during the therapeutic process, the expression levels of the autophagy-related mRNA and proteins were detected, along with the accumulation of autophagosome detected by MDC analysis in vitro and TEM in vivo. Increased levels of LC3 I/II, Atg5, and Beclin1 mRNA (*P* < 0.001 and *P* < 0.01 and *P* < 0.001), along with decreased levels of p62 mRNA (*P* < 0.001), were observed in the HO-1 group, which showed similar tendencies with the anisomycin group. In mice with shHO-1-MSC transplantation, the GCs’ autophagy was inhibited which presented as decreased levels of LC3 I/II and Beclin1 mRNA (*P* < 0.001 and *P* < 0.01) and decreased levels of p62 mRNA (*P* < 0.01), showing the similar tendency as the SP600125 administration (Fig. 5). For the aspect of the protein expression, HO-1-MSC administration can induce the autophagy in GCs showed as increased protein levels of LC3 I/II (*P* < 0.001), while shHO-1-MSCs transplantation reduced the autophagy presented as decreased levels of LC3 I/II, Beclin-1, and Atg5 (*P* < 0.01 and *P* < 0.05 and *P* < 0.05), and increased levels of p62 protein (*P* < 0.05), which were similar to the tendency in the SP600125 group. With the anisomycin treatment, the expression levels of LC3 I/II and Atg5 were increased (*P* < 0.001 and *P* < 0.05), while the expression levels of p62 protein were decreased (*P* < 0.01) (Fig. 6).

**Fig. 7:**
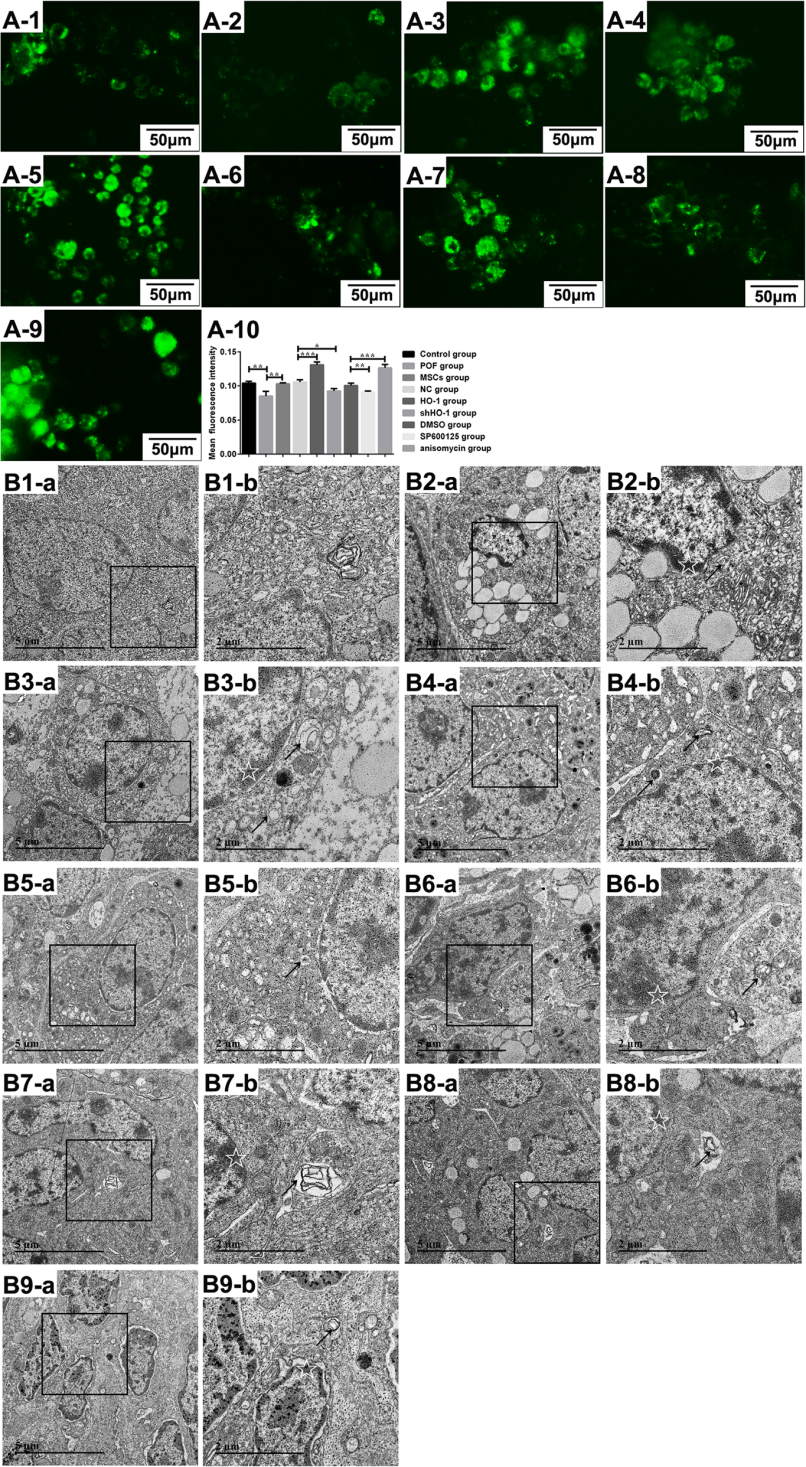
Induction of autophagy in GCs in response to HO-1/shHO-1-MSCs, treated with or without anisomycin or SP600125. **A-1**–A**-9** HN2-treated GCs co-cultured with UCMSCs transfected with HO-1/shHO-1 plasmids with or without the administration of SP600125 or anisomycin for 48 h in vitro. Representative fluorescent and phase-contrast images of the treated cells were shown. **A-1** GC group. **A-2** HN2 group. **A-3** MSC group. A**-4** NC group. **A-5** HO-1 group. A**-6** shHO-1 group. A**-7** DMSO group. **A-8** SP600125 group. A**-9** Anisomycin group. A**-10** Fluorescence intensity analysis of GCs in each group. B**1**–**B9** POF mice transplanted with HO-1/shHO-1-MSCs and administrated with SP600125 or anisomycin for 48 h and imaged by TEM in vivo. Representative images of cells are shown as B**1** control group. B**2** POF group. **B3** MSC group. **B4** NC group. **B5** HO-1 group. **B6** shHO-1 group. **B7** DMSO group. **B8** Anisomycin group. **B9** SP600125 group. White arrows indicate accumulation of autophagosome; black arrows indicate chromatin condensation. × 1900 (a), bar scale = 5 μm. × 4800 (b), bar scale = 2 μm. *P < 0.05, **P < 0.01, ***P < 0.001 vs POF, NC group, or DMSO group, separately. DMSO, dimethylsulfoxide; GCs, granulosa cells; HN2, chlormethine; HO-1, heme oxygenase-1; NC, empty vector; POF, premature ovarian failure; UCMSCs, umbilical cord mesenchymal stem cells

Additionally, comparing with the NC group, the fluorescence intensity of GCs stained with MDC was obviously increased (*P* < 0.001), while it was decreased in the shHO-1 group (*P* < 0.05, Fig. 7), which represents that HO-1 expressed in MSCs can enhance the autophagy ability of GCs in the therapeutic process. And with the anisomycin administration, the intensity of MDC was improved (*P* < 0.001), while the SP600125 treatment inhibited the intensity (*P* < 0.01). To further confirm the researches above, the structure changes of autophagosome in GCs were observed by TEM, which showed the accumulated number of lamellar structures and cytosolic autophagic vacuoles in the HO-1-MSC group or anisomycin group as the morphology in the control group (white arrows). On the contrary, shHO-1-MSCs or SP600125-treated cells were morphologically distinct with breakup of the cell into numerous bodies and with chromatin condensation (black arrows) (Fig. 7). Based on the results above, we can suggest that HO-1 expressed in UCMSCs help recover the ovarian function in POF mice through activating the JNK/Bcl-2 signal pathway-regulated autophagy.

### HO-1-transfected UCMSC transplantation upregulates the ratios of CD8^+^CD28^−^ T cells in POF mice

**Fig. 8:**
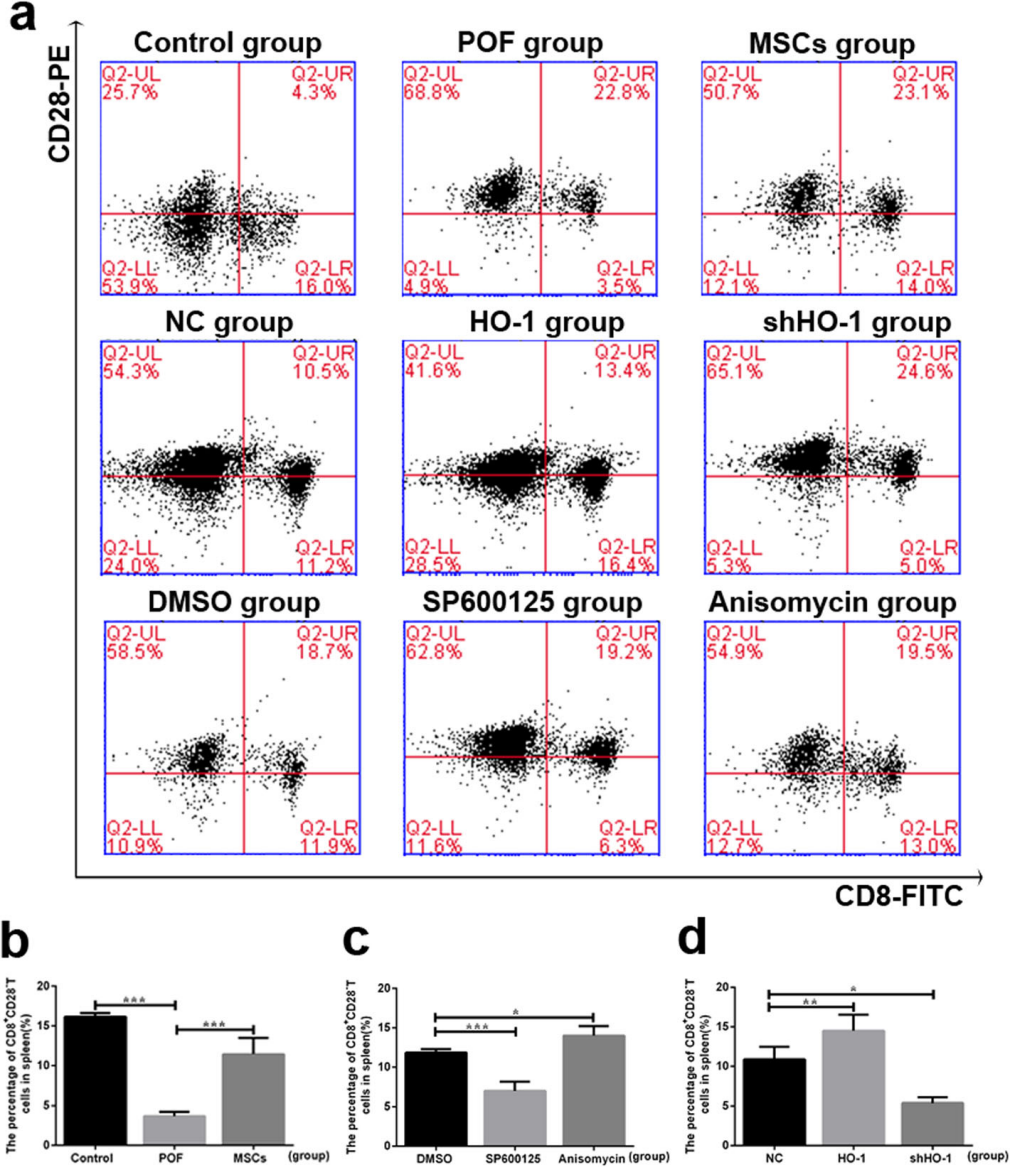
Effects of HO-1 gene expressed in the UCMSCs on ratios of CD8^+^CD28^−^ T cells with the administration of anisomycin or SP600125. **a** Representative flow cytometric plots for CD8^+^CD28^−^ T acquisition isolated from spleens in the nine groups, respectively. **b** The CD8^+^CD28^−^ T cells population comparison among the control group, POF group, and MSC group. **c** The CD8^+^CD28^−^ T cells population comparison among the DMSO group, SP600125 group, and anisomycin group. **d** The CD8^+^CD28^−^ T cells population comparison among the NC group, HO-1 group, and shHO-1 group. *P < 0.05, **P < 0.01, ***P < 0.001 vs DMSO, GC group, or NC group, separately. DMSO, dimethylsulfoxide; HO-1, heme oxygenase-1; NC, empty vector; POF, premature ovarian failure; UCMSCs, umbilical cord mesenchymal stem cells

To assess the essential role of the CD8^+^CD28^−^ T cells in the therapeutic process of POF mice receiving HO-1-MSC transplantation in vivo, spleen cells were separated and analyzed in each group (Fig. 8a). Comparing with the NC group, the frequency of CD8^+^CD28^−^ T cells was higher in the HO-1 group (*P* < 0.01) but lower in the shHO-1 group (*P* < 0.05), which means HO-1 gene can help promote the therapeutic efficiency of MSCs through upregulating the expression levels of CD8^+^CD28^−^ T cells (Fig. 8d). Moreover, much higher frequency of the cells was exhibited in mice with the administration of anisomycin (*P* < 0.05), and lower frequency was presented with SP600125 treatment (*P* < 0.001, Fig. 8c). Because IL-10 is one of the key predictors of inflammation produced by CD8^+^CD28^−^ T cells [36], it can be further detected to confirm the expression of CD8^+^CD28^−^ T cells during the therapeutic process. As showed in Fig. 3, with HO-1 transfection, the expression levels of IL-10 in mouse serum were elevated, showed as the similar tendency with the anisomycin group (*P* < 0.01, respectively); however, the expression were obviously reduced in mice with shHO-1-transfected MSC transplantation (*P* < 0.01) or with the administration of SP600125 (*P* < 0.01). Taken together, we can conclude that HO-1 helps improve the therapeutic role of MSCs through increase in the circulating of CD8^+^CD28^−^ T Cells.

## Discussion

Evidences have demonstrated that downregulated HO-1 levels were showed in abnormal metabolic states, and upregulated levels of HO-1 might suppress the inflammatory response and ameliorate metabolic disorders [34, 36]. However, it is still unknown if HO-1 expressed in UCMSCs is essential in recovering the ovarian function of POF mice receiving MSC transplantation. By comparing the outcomes of HO-1/shHO-1-transfected-UCMSC transplantation, we have confirmed that the ovarian function recovery is associated to the presence/absence of HO-1. Absence of HO-1 in UCMSC transplantation inhibits the GCs’ viability and activates their apoptosis in vitro. It also shows the decreased levels of E_2_ and AMH, the increased levels of FSH and LH in mice serum, the enhanced ovarian fibrosis, and the decreased functional follicles in vivo. However, the presence of HO-1 in UCMSC transplantation significantly recovers the ovarian function of POF mice with all these indices improved. Based upon these evidences, we can primary recognize that HO-1 expressed in UCMSCs played a critical therapeutic role in recovering the ovarian function of POF mice with UCMSC transplantation.

JNK can activate the autophagy through disruption of the Bcl-2/Beclin1 complex [29]. To reveal whether the JNK/Bcl-2 signaling pathway is involved in such recovery, we also used SP600125/anisomycin-treated mice to compare multiple ovarian functional indices. Similar outcomes are observed between mice with shHO-1-UCMSC transplantation and with SP600125 administration, as well as between mice with HO-1-UCMSC transplantation and with anisomycin administration. Results showed that both the administration of SP600125 and the transplantation of shHO-1-UCMSC inhibit the p-JNk expression, and the therapeutic efficiency was downregulated, which presented as decreased GCs’ viability and increased apoptosis in vitro, along with abnormal serum levels such as increased serum levels of FSH and LH, decreased levels of E_2_ and AMH, and the observation of ovarian fibrosis, declined number of functional follicles and inclined number of atretic follicles in vivo. HO-1-UCMSCs and anisomycin activate the JNK/Bcl-2 signaling pathway, and the changes above can be reversed.

As a cell degradation process, autophagy devours injured organelles and dysfunctional cytoplasm to maintain cell function [37], which is active in cells in physiological state, and can also be induced in response to cellular stress [38]. To figure out the critical role of the autophagy in the therapeutic process and its association with the JNK/Bcl-2 signal pathway and HO-1 gene, the expression levels of autophagy markers like LC3-II/I, Beclin1, p62, and Atg5 were measured in different conditions. LC3 conversion (LC3-I to LC3-II) reflects the progression of autophagy [39]; SQSTM1/p62 is widely used as a marker for autophagic flux [39–41], which is efficiently degraded by autophagy [42]. Atg5 is associated with phagophore formation [43], and beclin1 can direct the initiation phase and maturation-fusion phase [44]. Results showed that in mice with shHO-1-MSC transplantation or with the administration of SP600125, restrained autophagy in GCs were detected, including decreased expression levels of LC3-II, beclin1, and Atg5 but increased p62. Meanwhile, in mice transplanted with HO-1-MSCs or treated with anisomycin, the autophagy in GCs can be obviously activated, shown as increased levels of LC3-II, beclin1, and Atg5 but decreased p62. It is known that increased levels of autophagosomes can enhance its own formation and/or block the lysosomal processing [45]. In this study, decreased number of autophagosomes and receded intensity of fluorescence by MDC were observed in mice transplanted with shHO-1-MSCs or treated with SP600125, also with the observation of nucleolus shrinked and chromatin condensation. However, HO-1-MSC transplantation or anisomycin treatment can significantly increase the number of autophagosomes and numerous lamellar structures with cytosolic autophagic vacuoles with a relatively intact nucleus, and enhanced intensity of fluorescence by MDC. The positive and the negative results suggest the critical role of HO-1 in ovarian function by activating the JNK/Bcl-2 signaling pathway to regulate the autophagy.

Researches showed that the CD8^+^CD28^−^ T cells showed similar pleiotropic immunologic suppression to the traditional regulatory T cells [46]. In our study, the circulating CD8^+^CD28^−^ T cells inclined in mice with HO-1-MSC transplantation compared with that in the NC group, but declined in mice with shHO-1-MSC transplantation, suggesting that HO-1 played a critical role in the therapeutic process and a high level of CD8^+^CD28^−^ T cells is favorable for improving the therapeutic efficiency. IL-10 is one of the key predictors of inflammation produced by CD8^+^CD28^−^ T cells, which work on regulating T cell responses [47]. The upregulation level of IL-10 in the HO-1 group and downregulation level in the shHO-1 group further indicates that HO-1 played its role through upregulating the expression of CD8^+^CD28^−^ T cells.

## Conclusions

In summary, we have shown that HO-1 gene expressed in UCMSCs can help recover the ovarian function of POF mice with UCMSC transplantation through activating the JNK/Bcl-2 signal pathway-regulated autophagy and upregulating the circulating of CD8^+^CD28^−^ T cells. These findings provide a new target for the subsequent MSC-based therapies in humans in clinical.

## Data Availability

Data and materials in the current study are available from the corresponding author on reasonable request.

## Abbreviations

AMH: Anti-Müllerian hormone
CCK-8: Cell Counting Kit-8
DMSO: Dimethylsulfoxide
E_2_: Estradiol
FBS: Fetal bovine serum
FCM: Flow cytometry
FITC: Annexin-V-fluorescein isothiocyanate
FSH: Follicle stimulation hormone
FSHR: Follicle-stimulating hormone receptor
GC: Granulosa cell
HE: Hematoxylin and eosin
HN2: Chlormethine hydrochloride
IL-10: Interleukin-10
LH: Luteinizing hormone
PBS: Phosphate-buffered saline
POF: Premature ovarian failure
UCMSCs: Umbilical cord-derived mesenchymal stem cells
